# The relationship between social support in pregnancy and postnatal depression

**DOI:** 10.1007/s00127-022-02269-z

**Published:** 2022-04-22

**Authors:** Billie Lever Taylor, Selina Nath, Antoaneta Y. Sokolova, Gemma Lewis, Louise M. Howard, Sonia Johnson, Angela Sweeney

**Affiliations:** 1 Division of Psychiatry, Faculty of Brain Sciences, University College London, Maple House, 149 Tottenham Court Road, London, W1T 7NF UK; 2 Present Address: Population, Policy and Practice Research and Teaching Department, UCL Great Ormond Street Institute of Child Health, 30 Guilford Street, London, WC1N 1EH UK; 3 Lived Experience Advisory Group, University College London, Maple House, 149 Tottenham Court Road, London, W1T 7NF UK; 4 Health Services and Population Research Department, Institute of Psychiatry, Psychology and Neuroscience, Kings College London, De Crespigny Park, London, SE5 8AF UK

**Keywords:** Perinatal, Mental health, Social support, Postnatal depression, Service users, Quantitative

## Abstract

**Purpose:**

Lack of social support is considered a potential risk factor for postnatal depression but limited longitudinal evidence is available. Pregnancy, when women have increased contact with healthcare services, may be an opportune time to intervene and help strengthen women’s social networks to prevent feelings of depression postnatally, particularly for those at greatest risk. Our study examined the longitudinal relationship between social support in pregnancy and postnatal depression, and whether this is moderated by age or relationship status.

**Methods:**

We analysed data collected from 525 women from a diverse inner-city maternity population in England who were interviewed in pregnancy and again three months postnatally. Women provided sociodemographic information and completed self-report measures of depression (Edinburgh Postnatal Depression Scale) and social support (Social Provisions Scale).

**Results:**

Less social support in pregnancy was associated with postnatal depression, after adjusting for sociodemographic confounders and antenatal depression (Coef. = − 0.05; 95% CI − 0.10 to − 0.01; *p = *0.02). There was weak evidence of a moderating effect of relationship status. Subgroup analysis showed a stronger relationship between social support in pregnancy and postnatal depression for women who were *not* living with a partner (Coef. =  − 0.11; 95% CI − 0.21 to − 0.01; *p = *0.03) than for those who were (Coef. =  − 0.03; 95% CI − 0.09 to 0.02; *p = *0.28). Sensitivity analysis using multiple imputations to account for missing data confirmed the main results.

**Conclusions:**

Interventions that target social support in pregnancy have the potential to reduce depression postnatally. Future research should explore in greater detail which women would benefit most from which type of social support.

**Supplementary Information:**

The online version contains supplementary material available at 10.1007/s00127-022-02269-z.

## Introduction

The perinatal period, including pregnancy (the antenatal period) and up to one-year postnatally, is a time of transition for women and their families. Experiences of depression are common and suicide is a leading cause of maternal deaths in the year after pregnancy [1]. Finding ways to reduce feelings of depression and improve wellbeing among perinatal women is considered key.

The arrival of a new baby results in both continuities and changes in women’s family systems and social networks [2]. Women’s support networks have been found to become smaller and more homogeneous after having a baby [2] and women report feeling lonely during the transition to motherhood [3]. Relationship satisfaction in couples often declines in early parenthood [4]. Some women, such as those who are parenting alone, or are young or deprived, appear particularly likely to experience isolation and lack of social support [5, 6].

Social support refers to the ‘resources’ in a person’s interpersonal network that are available to them or that they feel able to draw on. While some researchers have argued that social support is unidimensional [7], most view it as multidimensional and there is overlap with related concepts like loneliness (distressing feelings of having inadequate relationships) and social isolation (a lack, or perceived lack, of social contact) [8].

Evidence suggests that social support becomes more important during critical transition periods like childbirth and can exert an increased influence on wellbeing [2]. Pregnancy could be a valuable time to intervene to help prevent depression by strengthening women’s social networks as it is a time when women have increased contact with the healthcare system. However, research into the impact of social support in pregnancy on the development of postnatal depression is limited. There is evidence that low social support is associated with increased postnatal depression [9, 10]. However, most studies have been cross-sectional meaning that directions of associations are unclear. Longitudinal associations between social support in pregnancy and depression postnatally have less commonly been explored, though some research exists. A longitudinal study in Australia [11] found that having less social support in pregnancy (measured by the Social Provisions Scale) is associated with an increased risk of postnatal depression. However, this was based on simple correlations, without adjustment for potential confounders, and included only a small cohort of 54 women all taking part in a trial of cognitive-behavioural therapy for depression.

A larger Australian study of 398 women identified that increasing antenatal social support has the potential to reduce rates of postnatal depression by up to 3% in women who report pre-existing mental health difficulties [12]. Similarly, a wider systematic review of antenatal risk factors for postnatal depression found that low social support in pregnancy is strongly associated with postnatal depression [13]. However, the studies reviewed had important limitations. Social support was defined and measured in different ways across studies, sometimes using just a single question or conflating it with related but separate concepts like social isolation. Individual studies often included ethnically and socially homogenous participants, unrepresentative of many populations around the world. And although efforts were made to exclude poor-quality studies, the review did not formally assess studies for quality despite a previous meta-analysis identifying that effect sizes for the relationship between social support and postnatal depression reduce as study quality increases [14]. In addition, while there are indications that interventions targeted at women at heightened risk of postnatal depression, such as those who are young or single, may be particularly valuable [15], there has been little attempt in past research to explore whether associations between social support and depression are moderated by factors such as age or relationship status.

Although there is, therefore, a growing body of evidence showing an association between social support and postnatal depression, more high-quality research is needed to explore the relationship longitudinally from pregnancy to postnatally, using validated measures of social support, with ethnically and socially diverse participants, and also investigating whether the association may be stronger for women at greater risk of postnatal depression.

The aim of the current study was to explore whether having lower social support in pregnancy is associated with the subsequent onset of postnatal depression, and whether this is moderated by relationship status or age in a cohort of women from a diverse inner-city hospital in England. This can help inform interventions for women, for example by providing information about the extent to which interventions that address social support or seek to strengthen women’s social networks during pregnancy are important.

## Method

### Participants and procedures

Participants for this study were drawn from the WEll-being in pregNancy stuDY (WENDY), which was part of a wider programme of research: ‘Effectiveness of Services for Mothers with Mental Illness’ (ESMI; NIHR RP-PG-1210–12,002) [16]. NHS ethics approval was obtained (ref: 14/LO/0075).

The WENDY study was originally designed to explore the effectiveness of two brief depression screening questions used by midwives in the UK (the ‘Whooley questions’ [17]). Pregnant women (*n =  *545) were recruited from a diverse maternity service in South East London. Sampling was stratified by whether women screened ‘positive’ or ‘negative’ for depression on the Whooley questions: all women screening ‘positive’ (i.e. depressed; 52.7%, *n =  *287) and a random sample of those screening ‘negative’ (i.e. non-depressed; 47.3%, *n =  *258) were invited to participate. Women were excluded if they were under 16, lacked the capacity to consent, or had a termination or miscarriage in their current pregnancy prior to their initial (baseline) interview. Full information on sampling strategy and recruitment can be found elsewhere [18].

Women were interviewed in early pregnancy (baseline interview, approximately 10–12 weeks gestation), and again in mid-pregnancy (approximately 28 weeks gestation). They were then followed up 3 months postnatally. Data were collected between November 2014 and June 2017. Written informed consent was obtained from all participants.

At the baseline (face-to-face) research interview during early pregnancy, participants completed a questionnaire pack containing questions about their sociodemographic status, mood and well-being. Women again completed questionnaires on their mood and well-being at follow-up interviews (either by telephone or face-to-face).

The current study analysed data on women’s responses to measures of social support and depression in early pregnancy (at baseline) and at 3-months postnatally.

### Involvement of people with relevant lived experience

The wider ESMI research programme included input from an advisory panel of women (and family members) with experience of perinatal mental health difficulties. For the current analysis, three women from this panel with relevant lived experience formed a lived experience advisory group (LEAG), grounding our analysis in experiential knowledge. This LEAG met four times, with discussions including the analysis plan, data interpretation and implications of the findings.

### Measures

#### Depressive symptoms

The Edinburgh Postnatal Depression Scale (EPDS) measures perinatal depressive symptoms [19]. It has been validated both antenatally and postnatally across different socioeconomic groups and languages [20–22]. The scale has 10 items, answered on a scale of 0–3. Higher scores reflect greater levels of reported depressive symptoms. A suggested cut-off of 13 + has been used to signify a major depressive episode, however, our LEAG discussed the limitations of using a single cut-off to represent the complexity and lived reality of depression. Therefore, in our analyses, we used the EPDS as a continuous measure, meaning that the experiences of women who reported some feelings of depression but scored below the cut-off point, were reflected in the findings. Using Cronbach’s α coefficient, the internal consistency of the scale was 0.90 at the early pregnancy baseline interview and 0.86 at the follow-up postnatal interview.

#### Social support

The Social Provisions Scale (SPS) is based on Weiss’s [23, 24] theory of social relationships, which Cutrona and Russell [25] operationalised into a measure of social support. Weiss argued that some aspects of wellbeing can only be met through supportive relationships, in particular six ‘social provisions’: attachment (emotional support from intimate partners), social integration (a sense of belonging to a group with shared interests), guidance (advice or information from trustworthy others), reliable alliance (the belief that social relationships can be relied on for support), reassurance of worth (recognition of competence and skills), and opportunity for nurturance (the sense that one is responsible for others). The SPS includes six subscales, based on these ‘social provisions’, and also produces a total continuous score (24 items rated on a scale of 1–4). It has been used both antenatally and postnatally and has good internal consistency, reliability and construct validity [25]. Higher scores reflect greater reported levels of social support. Using Cronbach’s α coefficient, the internal consistency of the scale was 0.93 at both the early pregnancy baseline and follow-up postnatal interviews. As four of the six subscales (guidance, social integration, attachment and reliable alliance) showed high levels of intercorrelation antenatally in our study (*r* > 0.70), we used only the total score.

#### Confounders

Women also provided socio-demographic information in their early pregnancy interview. We selected potential confounders a priori based on input from our LEAG and on prior literature on factors associated with postnatal depression (e.g. [26, 27]. We included employment status (employed or not), education (higher education or not), relationship status (living with a partner/husband or not), primiparity (first baby or not), age at baseline (i.e. early pregnancy, as a continuous measure), and ethnicity. We categorised ethnicity as (1) White English, Scottish, Welsh, Irish, Other; (2) Black African, Caribbean, Black British; (3) Asian, Asian British; (4) Mixed or Other Ethnicity. For the main analyses, we grouped women from categories 2, 3 and 4 in a single category. This was in part due to small numbers of participants from some ethnic groups, and also because early pregnancy (baseline) social support scores on the SPS were similar for women from Asian (*n =  *24, *M = *77.0, SD = 11.1), Black (*n =  *167, *M = *77.5, SD = 11.7) and Mixed or Other (*n =  *56, *M = *79.0, SD = 10.1) backgrounds, whereas they were higher for women from White backgrounds (*n =  *278, *M = *84.5, SD = 9.9).

### Data analysis

All analyses were conducted using Stata v.16. Social support and depressive symptom scores (on the SPS and EPDS, respectively) were described at the antenatal timepoint. Differences in antenatal SPS scores across key demographics were examined using independent group t-tests for binary variables and Pearson’s correlation coefficients for continuous variables. In an exploratory analysis, we examined changes in scores on the EPDS, from the antenatal to postnatal period, using a paired-samples *t *test.

We built the linear regression models to examine the association between social support (exposure) and depressive symptoms (outcome) in three stages. First, unadjusted regressions were carried out to investigate the association between antenatal social support (SPS) and postnatal depression (EPDS) (model 1). Second, multivariable regression models were run, adjusting for sociodemographic variables that were potential confounders (model 2). To investigate whether antenatal social support was associated with postnatal depression, independent of antenatal depression symptoms, antenatal EPDS scores were then added to the multivariable regression model (model 3). While EPDS scores were positively skewed (antenatally and postnatally), residuals from the regression appeared normally distributed.

Based on the prior literature outlined and informed by LEAG discussions, we additionally investigated whether relationship status or age modified the association. First, we calculated an interaction term for antenatal social support and each potential effect modifier. If there was evidence of statistical interaction, we presented the association between antenatal social support and depressive symptoms separately for each level of the effect modifier [28, 29]. We examined interaction terms with sociodemographic confounders and antenatal depression included as potential confounders. We also examined interaction terms without including antenatal depression, as we considered that antenatal depression could potentially be a mediator rather than a confounder of the relationship between antenatal social support and postnatal depression, resulting in over-adjustment [30].

### Missing data

**Fig. 1:**
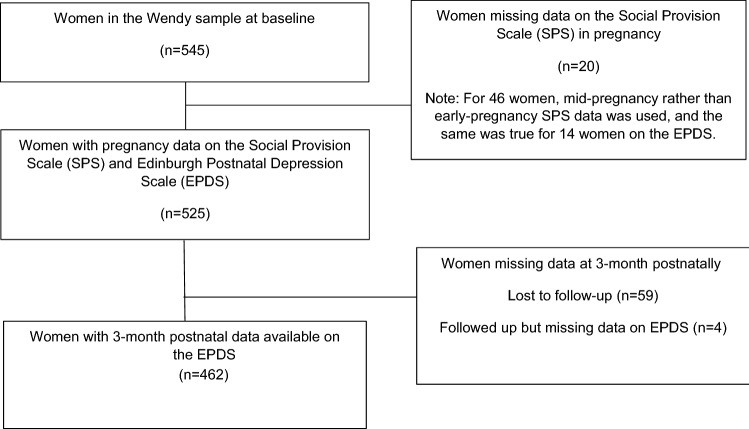
Flow-chart of participants through the study

In total, 545 women took part in the WENDY study at baseline (early pregnancy). A proportion of women (*n =  *66; 12.1%) had data missing at baseline on the SPS. Of these 66 women, 46 (69.7%) had complete mid-pregnancy SPS data available and this was used instead since we were primarily interested in exploring associations between social support antenatally (i.e. in pregnancy) and depression postnatally (i.e. after pregnancy), and the mean SPS score remained consistent at the early pregnancy (*M = *82.1, SD = 10.6) and mid-pregnancy (*M = *82.1, SD = 9.9) timepoints. This resulted in a total of 525 women with antenatal data on the SPS. Of these, just 14 women had missing data on the EPDS at baseline (5 of whom also had missing baseline data on the SPS). These 14 women all had complete mid-pregnancy EPDS data available, and this was used instead. The only confounder with missing data was employment status (where 2 women were missing data). At the 3-month postnatal time-point, 63 (12.0%) women had data missing on the EPDS. Most of these (*n =  *59; 93.7%) were missing all items on the EPDS, while the remainder (*n =  *4; 6.3%) had 1 item missing (see Fig. 1 for flow-chart of participants through the study).

We conducted a complete case analysis, including participants who had complete data on all variables in the analyses (this included 462 women with complete data on both the SPS antenatally and the EPDS postnatally and 460 women with complete data across potential confounders too). As a sensitivity analysis, we used multiple imputations with chained equations (MICE) to replace missing data on the outcome measure (i.e. the EPDS) and confounders (i.e. employment status, which was the only confounder with missing data). We assumed data were missing at random (i.e. that missing data were associated with observed data) and imputed 50 datasets. To impute the missing values, we used all variables included in our analyses as well as auxiliary variables (immigration status and whether a woman was late booking her initial midwife appointment). We re-ran analyses according to Rubin’s rules [31].

## Results

### Descriptive statistics

Table 1 shows characteristics at baseline (early pregnancy) for the 525 study participants. Women’s mean age was 32.9 years (SD 5.6). Just over half (53.0%; *n =  *278) were White, while nearly a third (31.8%; *n =  *167) were Black African, Black Caribbean or Black British. Just over half (52.8%; *n =  *277) had attended higher education and just under three quarters (73.1%; *n =  *384) were living with a partner. The median antenatal SPS score was 84 (IQR: 74–90) and on the antenatal EPDS was 7 (IQR: 4–12). There was evidence of a difference between mean antenatal ( *M = *8.4, SD = 6.1) and postnatal (*M = *6.5, SD = 5.2) EPDS scores: women reported feeling less depressed postnatally than antenatally, a decrease of 1.9 points on the EPDS (95% CI, 1.4–2.4; *p <  *0.001).

**Table 1:** Baseline (early pregnancy) characteristics of participants (*n =  *525)

Variable	Level	*N = *525
Age (at baseline)	Mean (SD)	32.9 years (SD 5.6)
Ethnicity	White	278 (53.0)
	Black African, Caribbean or Black British	167 (31.8)
	Asian or Asian British	24 (4.6)
	Mixed ethnicity	23 (4.4)
	Other ethnicity	33 (6.3)
Employment status	Employed	346 (66.2)
	Not employed	177 (33.8)
Higher education	No	248 (47.2)
(university degree or higher)	Yes	277 (52.8)
Relationship status	Living with partner	384 (73.1)
	Single/not living with partner	141 (26.9)
Any other children	No	261 (49.7)
	Yes	274 (50.3)
EPDS antenatal total score	Mean (SD)	8.6 (6.4)
	Median	7 (IQR: 4–12)
	Exceeding cut-off for major depression (13 +)	131 (25.0)
SPS antenatal total score	Mean (SD)	81.3 (11.1)
	Median	84 (IQR: 74–90)

All statistics are *n* (%) unless otherwise specified. There were 2 (0.4%) missing observations for ‘employment status’

As shown in Table 2, independent sample t-tests showed that scores on the SPS antenatally were lower (indicating lower social support) for women who were not living with a partner, not working, did not have higher education, and were of Black, Asian, Mixed or Other ethnic backgrounds. In each case, this was true across all SPS subscales, except in the case of ‘opportunity for nurturance’, which did not vary by employment status. There was no difference in antenatal SPS scores for first-time mothers compared to those with other children. A Pearson’s correlation coefficient showed a positive correlation between age and antenatal SPS scores (including across all SPS subscales), with younger women reporting less social support (*r* = 0.24, *p <  *0.01).

**Table 2:** Differences in antenatal social support scores across key demographic groups

Variable	SPS score	*N*	Mean difference	*P*
	*M* (SD)		(CI)	
Relationship status^a^	84.1 (8.8)	384	10.3 (8.0–12.6)	< 0.001
Living with a partner				
Not living with a partner	73.8 (12.9)	141		
Employment status^a^	83.5 (9.3)	346	6.2 (4.0–8.3)	< 0.001
Employed				
Not employed	77.3 (13.0)	177		
Education level^a^	84.1 (9.5)	277	5.9 (4.0–7.7)	< 0.001
Higher education				
No higher education	78.2 (11.9)	248		
Number of children^b^	81.2 (11.4)	261	− 0.4 (-2.3 to − 1.5)	0.71
One child				
More than one child	81.5 (10.7)	264		
Ethnicity^a^	84.5 (9.9)	278	6.7 (4.9–8.5)	< 0.001
White				
Black, Asian, Mixed or Other	77.8 (11.3)	247		

^a^Independent *t* test with unequal variances assumed

^b^Independent *t* test with equal variances assumed

### The association between antenatal social support and postnatal depression

In the unadjusted model, higher antenatal social support was associated with lower depressive symptoms postnatally. For every 1-point increase on the SPS, scores on the EPDS decreased by 0.16 points (95% CI, − 0.20 to − 0.11; *p <  *0.001) (Table 3, model 1).

**Table 3:** Associations between antenatal social support and postnatal depression

Exposure: SPS antenatal score	Coef	95% CI	*p*
Model 1^a^ (*N = *462)	− 0.16	− 0.20 to − 0.11	< 0.001
Model 2^b^ (*N = *460)	− 0.14	− 0.19 to − 0.10	< 0.001
Model 3^c^ (*N = *460)	− 0.05	− 0.10 to − 0.01	0.02

^a^Unadjusted

^b^Adjusting for maternal age (continuous in years), higher education (yes/no), employment status (working/not working), relationship status (living with a partner/not living with a partner), ethnicity (White/Black, Asian, Mixed or other), other children (yes/no)

^c^Adjusting further for depressive symptoms during pregnancy

After adjusting for sociodemographic factors (education, relationship status, employment status, age, primiparity, and ethnicity), evidence of the association between antenatal social support and depressive symptoms postnatally remained. For every 1-point increase on the SPS, EPDS scores decreased by 0.14 points (95% CI, − 0.19 to − 0.10; *p <  *0.001) (model 2).

After further adjusting for antenatal depressive symptoms on the EPDS, (model 3), the association between antenatal social support and postnatal depression was attenuated but remained. For every 1-point increase on the SPS, scores on the EPDS decreased by 0.05 points [(95% CI, − 0.10 to − 0.01); *p = *0.02].

### Moderation by age or relationship status

We found no evidence of a moderating effect of age on the association between antenatal social support and postnatal depression in model 1 (Coef. = − 0.00; 95% CI, − 0.01 to 0.01; *p = *0.63; unadjusted), model 2 (Coef. =  − 0.00; 95% CI, − 0.01 to 0.00; *p = *0.38; adjusting for confounding sociodemographic factors) or model 3 (Coef. =  − 0.00; 95% CI, − 0.01 to 0.00; *p = *0.28; adjusting also for antenatal depression).

There was weak evidence of a moderating effect of relationship status on the association between antenatal social support and postnatal depression. The interaction term for model 1 (unadjusted) was *p = *0.02 (Coef. =  − 0.11; 95% CI, − 0.20 to − 0.01), and for model 2 (i.e. adjusting for confounding sociodemographic factors) was *p = *0.04 (Coef. =  − 0.10; 95% CI, − 0.20 to − 0.00). When antenatal depression was added into the model (model 3), the evidence of interaction attenuated (Coef. =  − 0.04; 95% CI, − 0.13 to − 0.05; *p = *0.35). Subgroup analysis stratified by relationship status showed a stronger relationship between antenatal social support and postnatal depression for women who were *not* living with a partner than for women who were (Table 4). For women not living with a partner, the association between antenatal social support and postnatal depression remained after adjusting for sociodemographic factors and antenatal depressive symptoms (Coef. =  − 0.11; 95% CI, − 0.21 to − 0.01; *p = *0.03). For women who *were* living with a partner, there was no longer an association between antenatal social support and postnatal depression after adjusting for sociodemographic factors and antenatal depressive symptoms (Coef. =  − 0.03; 95% CI, − 0.09 to 0.02; *p = *0.28).

**Table 4:** Association between antenatal social support and postnatal depression stratified by relationship status

Exposure	Not living with a partner			Living with a partner		
	*n = *116			*n = *346		
	Coef.	95% CI	*p*	Coef.	95% CI	*p*
SPS antenatal score						
Model 1^a^	− 0.21	− 0.29 to − 0.13	< 0.001	− 0.10	− 0.16 to − 0.04	0.001
Model 2^b^	− 0.22	− 0.30 to − 0.13	< 0.001	− 0.10	− 0.16 to − 0.04	0.001
Model 3^c^	− 0.11	− 0.21 to − 0.01	0.03	− 0.03	− 0.09 to 0.02	0.28

^a^Unadjusted

^b^Adjusting for maternal age (continuous in years), higher education (yes/no), employment status (working/not working), relationship status (living with a partner/not living with a partner), ethnicity (White/Black, Asian, Mixed or other), other children (yes/no)

^c^Adjusting further for depressive symptoms during pregnancy

### Sensitivity analysis

In the multiple imputation analyses, results were broadly similar (see supplementary file). We found the same pattern where the association between antenatal social support and postnatal depression remained robust but was somewhat attenuated when antenatal depression scores were included in the model (Coef. − 0.05, 95% CI, − 0.10 to 0.01, *p = *0.03). Relationship status continued to show weak evidence of a moderating effect on the association (Coef. =  − 0.09; CI, − 0.18 to 0.00; *p = *0.06 in unadjusted model, and Coef. =  − 0.08; CI, − 0.18 to 0.01; *p = *0.08 when sociodemographic confounders were adjusted for). As with the complete case analysis, this was attenuated when antenatal depression was added into the model (Coef. =  − 0.03; CI, − 0.12 to 0.05; *p = *0.44). Age showed no evidence of a moderating effect (*p = *0.34 to 0.56).

Similar to the complete case analysis, subgroup analysis showed weak evidence of an association between antenatal social support and postnatal depression for women who were not living with a partner, even after adjusting for sociodemographic factors and antenatal depression (Coef. − 0.09, 95% CI, − 0.19 to 0.01, *p = *0.08), but not for those who were living with a partner (Coef. − 0.03, 95% CI, − 0.09 to  0.02, *p = *0.24).

## Discussion

We explored the association between antenatal social support and postnatal depression in a diverse cohort of women recruited from an inner-city hospital in England. We found that having less antenatal social support was associated with a greater subsequent risk of postnatal depression (at three months postnatally). This association remained after adjusting for potential sociodemographic confounders, and was attenuated but still robust after further adjusting for antenatal depression. These findings extend a growing body of evidence that antenatal social support plays an important role in relation to postnatal depression [10, 13], independently of concurrent antenatal depression [12].

A novel aspect of our work was that we also found weak evidence that relationship status moderated the association between antenatal social support and postnatal depression: social support in pregnancy was lower among women not living with a partner and the effect size for the association between lower antenatal social support and increased postnatal depression was larger for these women than for those who *were* living with a partner. Previous research has identified that mothers parenting alone may face unique challenges [5, 6, 32] and that reporting a lack of support from a partner may be especially influential in the development of postnatal depression [12]. Our findings suggest that having strong emotional and practical social support perinatally may be particularly important in preventing postnatal depression for mothers not living with a partner and this would merit further research.

Our findings suggest that maternity and mental health professionals should be made aware that low social support is a likely risk factor for postnatal depression. While midwives in the UK are now expected routinely to ask women about feelings of depression during pregnancy, it may be helpful also to ask about available family and social support, and for this to be a focus of wider public health initiatives too. Social support is increasingly seen as a priority area when supporting people experiencing mental distress [33] and, as outlined, pregnancy may be an opportune moment to intervene. Our LEAG highlighted the importance of peer support at this time, and there is growing evidence that peer support is effective for perinatal women experiencing depression [34]. Our findings indicate that developing and evaluating perinatal interventions that strengthen women’s interpersonal and social networks may help prevent or reduce postnatal depression.

However, both our LEAG and previous research have also emphasised the need for interventions to be sensitive to the ways in which factors like age, socioeconomic status, and relationship status may affect women’s experiences [15, 32]. Our finding that antenatal social support and postnatal depression appear more strongly related for women not living with a partner than for those who are adds weight to the idea that needs and experiences may differ for different women, and future research should investigate this further. As outlined, there is some indication that interventions targeted at women at greater risk of postnatal depression, such as those who are young or single, may produce greater benefits [15], but further research is needed. While we did not find evidence of a moderating effect by age, few women under 25 participated in the study, so it was not possible to determine fully whether the needs of younger mothers may be different.

Our study had other limitations. Attrition is a common difficulty with cohort studies and was apparent in ours, but sensitivity analyses using multiple imputations produced similar findings, suggesting our results were unlikely to be due to selection bias. While we cannot be certain that data were solely missing-at-random (as we cannot rule out missing not at random) we were able to identify several variables associated with missingness, supporting the missing-at-random assumption.

As outlined, we used a longitudinal design and adjusted for antenatal depression in our data to account for the possible confounding effect of this variable. However, it is possible that antenatal depression could mediate the association between social support and postnatal depression rather than confound it, and this could have resulted in underestimating the association [30].

The use of self-report measures could also have introduced measurement error, although the measures we used have generally shown good sensitivity and specificity. Nevertheless, it is possible that the Social Provisions Scale (SPS) may have limitations in the perinatal context. Firstly, the ‘opportunity for nurturance’ subscale of the SPS results in a higher score if greater opportunity to nurture others is reported. But this could be misleading in the context of childbirth where increased opportunities to nurture another may not necessarily be experienced as increasing feelings of social support. Secondly, as outlined four of the six subscales were highly intercorrelated, suggesting significant overlap. It would be helpful for future research to seek to replicate our findings using different measures of social support and/or to validate the SPS further in perinatal populations. Previous research suggests that different components of social support may have a differential influence on postnatal depression, with support from one’s partner or own mother found to be more influential than support from friends and other family [10, 12]. This would also merit further investigation, as the Social Provisions Scale does not make a distinction between partners, family members or close friends. Greater consideration of the influence of relationship conflict or violence in the perinatal period would also be valuable, as this is also not a focus of the SPS [35].

Despite these limitations, our study is novel and had some key strengths. We interviewed an ethnically and socially diverse group of women, representative of the target inner-city population [18]. The prospective study design allowed us to make inferences about the direction of associations. While future research would benefit from the inclusion of more women from different ethnic groups (e.g. Asian or Arab), we used interpreters which enabled us to include women who did not speak English and who are often excluded from cohort studies: our LEAG considered the use of interpreters as critical in broadening involvement. Input from our LEAG also helped ensure wider experiential knowledge shaped our interpretation of the data and that multiple standpoints, both experiential and theoretical, informed our understanding of the findings.

In conclusion, in this study lower levels of antenatal social support were associated with increased postnatal depression, independent of sociodemographic factors and after adjusting for antenatal depression. Interventions that target social support may help reduce distress by strengthening women’s support networks. However, future research should explore in greater detail any variations across different groups of women, such as those not living with a partner and young and/or deprived mothers.

## Commentary by lived experience group member Eleanor O’Sullivan

I participated in this study as a Lived Experience advisor along with two other mothers who have experienced loneliness in the perinatal period. We met on Zoom but were given sufficient space and time to hear one another and respond to these findings in a lively and in-depth way. I think I can speak for all of us when I say that we appreciated the breadth of the study, taking in as it did women from different cultures, ages, relationship and work statuses. I was pleased that this was mirrored in our small group of advisors as well. Personally, I found it both challenging and cathartic to re-examine my feelings of loneliness, especially during Covid when social interactions were curtailed once more.

The study has revealed that the higher the antenatal social support is, the lower depressive symptoms are postnatally. The recommendation running through the report is to make maternity and mental health professionals aware of this to use the contact mothers may have with these services during the antenatal period to help build a strong social network that will already be in place when the baby is born. I really wish I had been advised to put some social support in place before I had my baby as I think that would have made a big difference to the depth and length of my depressive symptoms. Asking women which type of social intervention they would like is also very important. A mother I have met recently, who does not live with a partner, has to work full time Monday–Friday to pay the bills so would like social contact at the weekend. The mother and baby groups in her area are designed around mothers on maternity leave and therefore meet during the week, the weekend being set aside to spend with their partners. The inaccessibility of these groups for this mother has further fuelled her sense of social isolation.

As they have been identified as ‘influential’ support figures, it would also be good to have a closer look at the experience of women living without the support of a partner, or their own mother/mother figure, or both. Peer support came up a number of times in our advisory group meetings. Is there an opportunity here to link mums experiencing loneliness with other people who feel the same way—other lonely mothers is an obvious example but how about including older people who live on their own, for example?

## Supplementary Information

Below is the link to the electronic supplementary material.

Supplementary file1 (DOCX 16 KB)

## Data Availability

Full study protocol (approved by the Research Ethics Committee) and patient-level data are available from Chief Investigator Professor Louise Howard (louise.howard@kcl.ac.uk). Consent for data sharing was not obtained, but the presented data are anonymised and the risk of identification is very low.
