# Supplementary material for: The relationship between social support in pregnancy and postnatal depression
Source: Soc Psychiatry Psychiatr Epidemiol. 2022 Apr 22;57(7):1435–44. doi: 10.1007/s00127-022-02269-z (PMC9246777; doi:10.1007/s00127-022-02269-z)
Supplement: Supplementary file 1 — Supplementary file1 (DOCX 16 KB) [file 127_2022_2269_MOESM1_ESM.docx]

**Supplementary file**

**Sensitivity analysis of the association between antenatal social support and postnatal depression using multiple imputation (N=525)**

|  |  | | |
| --- | --- | --- | --- |
|  |  | | |
| **Exposure: SPS antenatal score** | Coef. | 95% CI | p |
| Model 1^a^ | -0.16 | -0.20 to -0.12 | <.001 |
| Model 2^b^ | -0.15 | -0.20 to -0.10 | <.001 |
| Model 3^c^ | -0.05 | -0.10 to -0.01 | 0.03 |

a Unadjusted

b Adjusting for maternal age (continuous in years), higher education (yes/no), employment status (working/not working), relationship status (living with a partner/not living with a partner), ethnicity (White/Black, Asian, Mixed or other), other children (yes/no)

c Adjusting further for depressive symptoms during pregnancy

**Sensitivity analysis of the association between antenatal social support and postnatal depression stratified by relationship status (n=525)**

|  | **Not living with a partner** | | | **Living with a partner** | | |
| --- | --- | --- | --- | --- | --- | --- |
|  | n=141 | | | n=384 | | |
| **Exposure** | Coef. | 95% CI | p | Coef. | 95% CI | p |
| SPS antenatal score |  |  |  |  |  |  |
| Model 1^a^ | -0.20 | -0.27 to -0.12 | <.001 | -0.11 | -0.17 to -0.06 | <.001 |
| Model 2^b^ | -0.20 | -0.29 to -0.12 | <.001 | -0.11 | -0.17 to -0.05 | <.001 |
| Model 3^c^ | -0.09 | -0.19 to 0.01 | 0.08 | -0.03 | -0.09 to 0.02 | 0.24 |

a Unadjusted

b Adjusting for maternal age (continuous in years), higher education (yes/no), employment status (working/not working), relationship status (living with a partner/not living with a partner), ethnicity (White/Black, Asian, Mixed or other), other children (yes/no)

c Adjusting further for depressive symptoms during pregnancy
